# Towards a Nitinol-Based Microfabricated Approach to Repair Long-Gap Esophageal Atresia

**DOI:** 10.3390/mi17050582

**Published:** 2026-05-07

**Authors:** Ana R. Domingues, Joana Silva, Lara Teles, Bernardo S. Dores, Alice Miranda, Sofia Martinho, Jorge Correia-Pinto, Bruno Esteves, Eliana M. F. Vieira, Manuel F. Silva, José H. Correia, Sara Pimenta

**Affiliations:** 1 CMEMS-UMinho, University of Minho, 4800-058 Guimarães, Portugal; id10557@alunos.uminho.pt (A.R.D.); pg56131@alunos.uminho.pt (J.S.); larateles75@gmail.com (L.T.); bernardo.dores@cmems.uminho.pt (B.S.D.); bruno.esteves@dei.uminho.pt (B.E.); evieira@dei.uminho.pt (E.M.F.V.); fsilva@dei.uminho.pt (M.F.S.); higino.correia@dei.uminho.pt (J.H.C.); 2 Life and Health Sciences Research Institute (ICVS), School of Medicine, University of Minho, 4710-057 Braga, Portugal; alicemiranda@med.uminho.pt (A.M.); sofiamartinho@med.uminho.pt (S.M.); jcp@med.uminho.pt (J.C.-P.); 3 ICVS/3B’s—PT Government Associate Laboratory, Braga/Guimarães, Portugal; 4 Pediatric Surgery Department, Hospital de Braga, Braga, Portugal; 5 LABBELS—Associate Laboratory, Braga/Guimarães, Portugal

**Keywords:** shape memory alloy, electrical tests, FEM simulations, NiTi thin-films, sputtering deposition

## Abstract

Long-gap esophageal atresia is a congenital anomaly that requires challenging repair procedures that are often associated with complications. This work proposes the use of nitinol to repair long-gap esophageal atresia. A first proof-of-concept with commercial nitinol is presented. Experimental tests and simulations were performed, including the application of electrical currents to promote nitinol heating and consequent contraction, tensile tests, chemical analysis, and ex vivo tests using porcine esophageal tissues. A preliminary experiment is also presented regarding NiTi sputtering deposition and the morphological, chemical, and crystallographic analysis of the thin-films, featuring the implementation of a microfabricated solution. The experimental electrical tests were in accordance with the simulations. The nitinol electrical resistance (0.8–1.5 Ω) decreased as its temperature increased (20–60 °C) with the application of electrical current (<1 A), which was consistent with the experimental Seebeck coefficient (6.49 ± 0.46 µV/K). The measured forces (6.5 N at 45 °C) are also in accordance with traction sutures. Chemical analysis revealed a passive titanium dioxide layer reported for nitinol. Regarding the ex vivo tests, the average nitinol final length was 28.5 mm, below 30 mm (threshold for long-gap esophageal atresia). Finally, preliminary results from NiTi sputtering confirmed well-controlled deposition and the viability of scaling this approach, opening new avenues for nitinol-based biomedical devices.

## 1. Introduction

Esophageal atresia (EA) is a congenital anomaly characterized by a disruption of the continuity of the esophagus. The prevalence of this anomaly is 1 per 2500 to 4500 live births. In more than 90% of the cases, the anomaly is associated with a tracheoesophageal fistula (TF), where the esophagus is connected to the trachea. According to the Gross classification system, there are 5 types of EA: type A, with a frequency of 7%, which is a pure EA without TF; type B, with a frequency of 2%, which is an EA with a proximal TF; type C, with a frequency of approximately 85%, which is an EA with a distal TF; type D, with a frequency lower than 1%, which is an EA with proximal and distal TF; and type E, with a frequency of 4%, characterized by an H-type fistula [1].

This anomaly can be prenatally suspected (in 10 to 40% of cases) or diagnosed in the immediate postnatal period. The prenatal suspicion is based on the identification of polyhydramnios, a small/absent stomach, and a dilated upper esophageal pouch. Both EA type A and type B are more frequently prenatally diagnosed than the other types, due to the absence of a distal fistula, hence a small/absent stomach. Obstetric ultrasound is used as the standard method to suspect EA prenatally, followed by other imaging techniques, such as fetal magnetic resonance imaging. The postnatal period is clinically characterized by excessive saliva accumulation (bubbles in the mouth and nose), recurrent cough, cyanosis, and respiratory difficulties after feeding attempts. The diagnosis is confirmed by chest radiography, in which the non-progression of a nasogastric or orogastric probe is observed [1,2,3].

EA treatment depends on its type and the distance between the upper and lower esophageal segments. In most cases, ligation of the TF and EA repair with a primary anastomosis should be performed in the first days of life. For several years, thoracotomy was used, but thoracoscopy has been emerging as a new gold standard since it is less invasive and is thought to improve long-term outcomes [4,5].

Long-gap EA is usually defined when the distance between the esophageal segments is larger than 30 mm or more than 2 vertebral bodies. Another possible definition, suggested by some authors, is to consider purely EA types A or B as long-gaps, meaning that long-gap EA does not have a distal TF but may or may not have a proximal TF. For practical and surgical purposes, a long-gap EA occurs when the defect is too large to be repaired by primary anastomosis, due to the complexity of bridging the substantial gap between the esophageal segments. Long-gap EA is associated with a higher morbidity, and there is no consensus about its ideal treatment approach [6]. Traditional techniques are often associated with high rates of complications (such as leakage, gastrointestinal reflux, and stenosis) and considerable morbidity. Such techniques include delayed primary anastomosis [7], the Foker technique [8] (external or internal stretching), and esophageal replacement [9]. Esophageal approximation and/or anastomosis using magnets (magnamosis) [10,11] has emerged, but it is also reported to be associated with high rates of complications, particularly stenosis. Recently, a more refined method involving thoracoscopic esophageal elongation using internal traction sutures has been developed, typically performed in a two-stage process [12].

**Figure 1:**
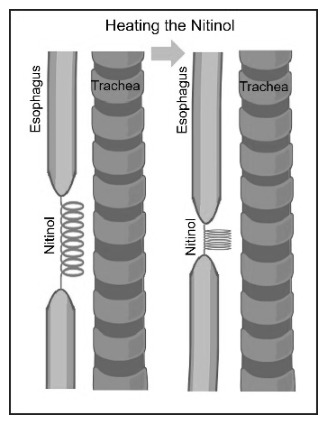
Overview of the use of nitinol to repair long-gap esophageal atresia. Heating the nitinol leads to its contraction and, consequently, to the approximation of the esophageal segments.

As previously stated, long-gap EA is complex and challenging to repair. No procedure is considered ideal, although preserving the native esophagus is widely regarded as the best approach. Thus, developing alternative methods is crucial to improving patient outcomes. This work proposes the use of a shape memory alloy, specifically nitinol (NiTi), to repair long-gap EA. The innovative concept involves the use of nitinol to gradually approximate the esophageal segments, allowing effective anastomosis. An overview of this innovative approach is shown in Figure 1. The research presented here provides the first proof-of-concept for this potential solution, using commercial nitinol coil springs, as well as a simulation model to preview the thermoelectrical performance of the commercial nitinol. In future work, we intend to microfabricate a nitinol spring more suitable for this biomedical application, concerning the electrical, thermal, and mechanical requirements. Some preliminary research work through the microfabricated solution is also presented and discussed in this paper.

## 2. Materials and Methods

### 2.1. Shape Memory Alloy

Nitinol is an alloy with a near-equiatomic mixture of nickel (Ni) and titanium (Ti). It has optimal mechanical characteristics and biocompatibility and exhibits the shape memory effect. This effect is the capacity to return to its original form after deformation when the temperature rises. The application of nitinol in the biomedical area is well-established. The shape memory effect of nitinol is achieved due to its two crystallographic phases: martensite and austenite. An elastic martensite phase exists at low temperatures and a rigid austenite phase at higher temperatures. The temperature at which the conversion occurs is named the transformation temperature and can be modified by changing the compositions of Ni and Ti [13,14,15,16,17].

This study used commercial nitinol 1-way memory coil springs from Nexmetal Corporation [18]. The commercial nitinol can be deformed when cold (retaining a deformed state after the removal of the external force) and returns to its original shape when heated. The main features of the coil springs are: wire diameter of 0.75 mm, outer diameter of 6.5 mm, fully contracted (hot) length of 16 mm, fully extended (cold) length of 50 mm, and transformation temperature of 45 °C. According to the literature, as the transformation temperature of the used commercial nitinol is 45 °C, it is expected that the composition of Ni is higher than 50 at.%, specifically between 50.59 and 50.8 at.% [19].

### 2.2. Experimental Tests

To observe and investigate the behavior of the commercial nitinol, a simple initial experiment was conducted in heated water. The nitinol was immersed in water at 45 °C (its transformation temperature). Immediately before the immersion, the nitinol was deformed to a length of 50 mm, in the cold martensite phase. After immersion in the heated water, the final length of the nitinol was measured after its contraction.

Electrical tests were also performed with the commercial nitinol, in which an electrical current was applied to induce Joule heating. An electrical current between 0.2 A and 0.9 A was applied. The setup included a DC power supply (Keysight E3648A, Santa Rosa, CA, USA), a digital multimeter (Keysight 34410A, Santa Rosa, CA, USA), and an infrared camera (Optris PI 400i, Berlin, Germany). Thus, it was possible to measure the maximum and average temperature of the nitinol, as well as its electrical resistance during the tests. Once more, immediately before the applied current, the nitinol was deformed to a length of 50 mm, in the cold martensite phase.

The electrical resistance of the commercial nitinol was also measured using the Kelvin method [20], considering the nitinol fully contracted (16 mm) and fully extended (50 mm) at room temperature, and using a digital multimeter (Keysight 34410A).

The Seebeck coefficient (S) of the commercial nitinol was measured using a custom-built setup and based on the “two-probe” method [21]. A temperature gradient (∆T) was generated along the nitinol, using a DC source (Yokogawa 7651, Tokyo, Japan). The resulting thermovoltage (∆V) was measured using a digital multimeter (Keysight 34410A). The measurements were performed 5 times for the nitinol, and a plot of ∆V vs. ∆T was obtained. S value (slope) and error were obtained by linear least squares fitting. Measuring the Seebeck coefficient in nitinol is important for characterizing its electrical transport behavior, directly influencing its heating response. Additionally, this parameter validates the temperature-dependent resistance changes and enhances the thermal-electrical models used to predict nitinol behavior, which are essential for reliable applications.

To evaluate the contraction force of the commercial nitinol during thermal activation, tensile tests were also performed using a universal testing machine (Shimadzu Autograph AG-IS, Kyoto, Japan) combined with an infrared camera for thermal monitoring (Optris PI 400i). The test had a duration of 5 min, consisting of Joule heating and subsequent passive cooling to room temperature. To perform this test, a high electrical current was applied (1.3 A) for approximately 155 s.

Finally, the surface chemical composition of the commercial nitinol was assessed by EDS (energy dispersive X-ray spectroscopy) using an EDAX Pegasus X4M system (Ametek EDAX, Mahwah, NJ, USA).

### 2.3. FEM Simulations

Simulations were also performed to validate the experimental electrical tests performed on the commercial nitinol. The COMSOL Multiphysics 5.3 software was used to obtain the electrical resistance of the nitinol and to study the temperatures reached during the application of an electric current at room temperature.

The first step was to design the commercial nitinol in the software and to obtain its electrical resistance, when fully extended (50 mm) and fully contracted (16 mm), both at room temperature. The nitinol geometry parameters are shown in Table 1. The nitinol physical parameters in the martensite phase are presented in Table 2 [22,23,24,25,26,27].

Secondly, the behavior of nitinol under an applied electrical current was investigated, with an emphasis on the simulation of the resulting temperature evolution and electrical resistance. The nitinol geometry parameters presented in Table 1 were used, considering the fully extended nitinol (50 mm). The nitinol physical parameters in the austenite phase are presented in Table 3 [22,23,24,25,26,27,28,29]. An electrical current was applied from 0.1 A to 1.0 A, with steps of 0.1 A.

Regarding the nitinol’ reached temperature, the heat flux was considered due to the convective heat transferring from the object to the room environment. The reference room temperature ($T_{ref}$) was set to 293.15 K (20 °C), and the convective heat transfer coefficient (h) was calculated to simulate heat exchanges between nitinol and the environment. This coefficient depends on the fluid motion (air in contact with the nitinol), thermal diffusion, and geometry [30]. The heat flux (q″) represents the rate per unit area [31]:

(1) $$q^{\prime \prime }=h(T_{s}-T_{ref})$$

where $T_{s}$ is the nitinol surface temperature, and h changes due to flow conditions and fluid (air) properties. The coefficient h can be calculated based on the Nusselt number (Nu), as shown below [31]:

(2) $$Nu=\frac{hD}{k}$$

where D represents the nitinol wire diameter (0.75 mm) and k represents the air thermal conductivity (0.025 W/mK [32]). Nu also varies as a function of the Reynolds number (Re) and the Prandtl number (Pr) [31]:

(3) $$Re=\frac{UD}{v}$$

(4) $$Pr=\frac{v}{a}$$

where U is the air velocity (it was considered 0.09 m/s [33]), v is the air kinematic viscosity (1.516 × 10^−5^ m^2^/s [32]), and a is the thermal diffusivity. Considering the contact with air at 20 °C, the Pr is approximately 0.7309 [32]. Churchill and Bernstein proposed a single comprehensive equation that covers a wide range of Re and Pr. The equation is applied to flow across cylinders and spheres [31]:

(5) $$Nu=0.3+\frac{0.62{Re}^{1/2}{Pr}^{1/3}}{{\left[1+{\left(0.4/Pr\right)}^{2/3}\right]}^{1/4}}{\left[1+{\left(\frac{Re}{282000}\right)}^{5/8}\right]}^{4/5}$$

Therefore, it was possible to obtain Re, Nu and h, at 4.4525, 1.3376, and 44.837 W/m^2^K, respectively.

Electrical resistance was considered using an equation that describes the variation in the conductivity of nitinol (σ) with the temperature [34]:

(6) $$\sigma =\frac{\sigma_{0}}{\left[1+\alpha \left(T_{s}-T_{ref}\right)\right]}$$

where $\sigma_{0}$ is the initial electrical conductivity, and α is the temperature coefficient of resistivity (see Table 3). A negative α was considered, since the experimental results show that resistance (and resistivity) decreases with increasing temperature. The experimentally obtained Seebeck coefficient was also considered in the simulations.

### 2.4. Ex Vivo Tests

**Figure 2:**
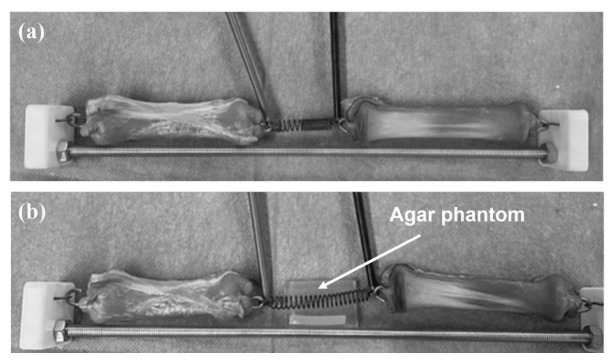
Ex vivo tests with porcine esophageal tissues and the commercial nitinol, without agar phantom (**a**) and with an agar phantom (**b**).

Ex vivo tests were performed with porcine esophageal tissues and the commercial nitinol. They were performed with thermal monitoring, using an infrared camera (Optris PI 400i). The tests were initiated with the removal of a 50 mm segment of the esophagus. The nitinol was then sutured to the open ends of the esophageal segments. Custom-fabricated PLA (polylactic acid) connectors were implemented to achieve better tension distribution on the esophagus tissues, as shown in Figure 2. The first test was conducted using the porcine esophagus (Figure 2a). The second test used the porcine esophagus and an agar phantom in proximity to the nitinol (Figure 2b), mimicking surrounding tissues in a real scenario application. For the two tests, different electrical current values were applied to the nitinol, between 0.5 A and 0.9 A. The current was adjusted until the average temperature of the nitinol was close to 45 °C. The average and maximum temperature of the nitinol, as well as the maximum temperature of the agar phantom (in the second test), were registered. Additionally, the time to reach the maximum contraction of the nitinol, the minimum length of the nitinol (corresponding to maximum contraction), and the final length of the nitinol were registered. The final length was obtained approximately 5 min after stopping the application of electrical current.

### 2.5. Microfabricated Approach: NiTi Thin-Films Sputtering Deposition

New nitinol spring designs for the proposed medical application are also being investigated. These consist of microfabricated custom nitinol springs for the repair of long-gap EA, designed and microfabricated with dimensions and transformation temperature optimized for newborns. Here, we present the first steps towards the final goal.

NiTi films were deposited on silicon substrates by DC magnetron sputtering, using a NiTi target (PhotonExport 48 at.% Ni and 52 at.% Ti). Table 4 presents the main deposition parameters used in several depositions to obtain different thin-films (1 to 5). Several parameters were tested, varying the argon gas flow, the power, and the substrate–target distance. The deposited thin-films were analyzed with SEM (scanning electron microscopy), using a NanoSEM–FEI Nova 200 (Thermo Fisher Scientific, Waltham, MA, USA), and EDS, using an EDAX Pegasus X4M system. The thin-film 2 was also annealed in vacuum (maximum temperature of 550 °C, D-well of 10 min, and heating/cooling ramp of 30 °C/min) to promote NiTi crystallization after deposition. Thin-films 1 and 2 were also analyzed with XRD (X-ray diffraction), using a Bruker D8 Discover system (Billerica, MA, USA), to compare their crystallographic structure.

## 3. Results and Discussion

### 3.1. Thermal Activation with Water Immersion

**Figure 3:**
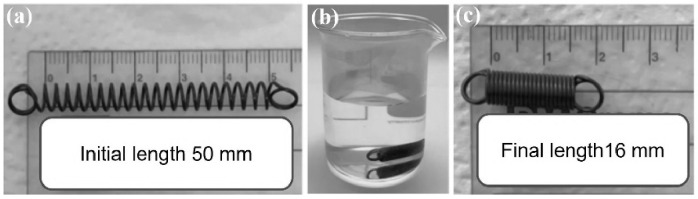
Test in heated water at 45 °C, showing the initial length of the commercial nitinol (**a**), its immersion in heated water (**b**), and its final length after contraction (**c**).

Figure 3 shows the result obtained after immersion of the commercial nitinol (deformed to a length of 50 mm) in water at 45 °C. A rapid contraction of the nitinol was observed when its surface was in contact with the heated water. The nitinol contracted rapidly until its minimum length (16 mm).

### 3.2. Electrical Thermal Activation

**Figure 4:**
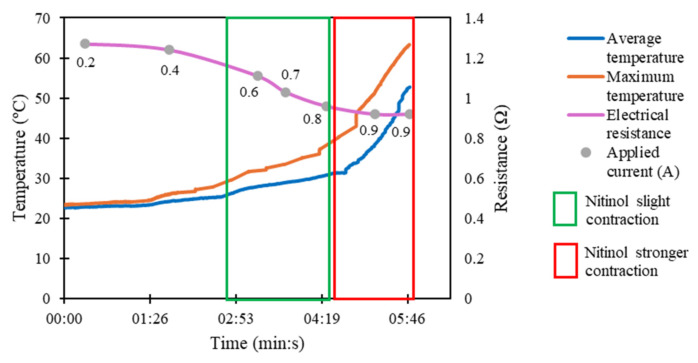
Experimental study of Joule heating of the commercial nitinol.

The heating of the commercial nitinol was promoted through the application of electrical currents, between 0.2 A and 0.9 A. Figure 4 presents the experimental results obtained. The electrical resistance decreases as the temperature increases. This observation agrees with the reported results of T. Kakeshita et al. [29] for nitinol alloys with Ni content higher than 50 at.%. The nitinol starts its contraction when its maximum temperature is around 30 °C. Moreover, its contraction becomes stronger and more effective when the applied electrical current is 0.8 A, and its maximum temperature is around 40 °C, which closely agrees with the transformation temperature of 45 °C (austenite finish temperature), specified by the manufacturer.

The electrical resistance of the commercial nitinol was also obtained using the Kelvin method. A resistance of 0.796 Ω was obtained for the fully contracted nitinol (16 mm of length), and of 0.785 Ω for the fully extended nitinol (50 mm of length), both at room temperature.

**Figure 5:**
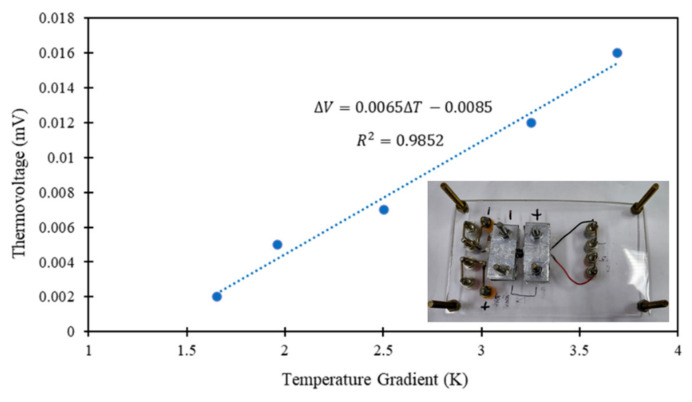
Experimental Seebeck coefficient for the commercial nitinol.

Finally, the obtained Seebeck coefficient for the commercial nitinol was 6.49 ± 0.46 µV/K, as can be seen in Figure 5, meaning a hole-dominated carrier transport. The positive Seebeck coefficient in nitinol is related to both the martensite-to-austenite phase transition and the alloy composition (with Ni content slightly higher than 50 at.%). This result is also in agreement with the very recent study reported by Lünser et al. [35].

### 3.3. Tensile Test

**Figure 6:**
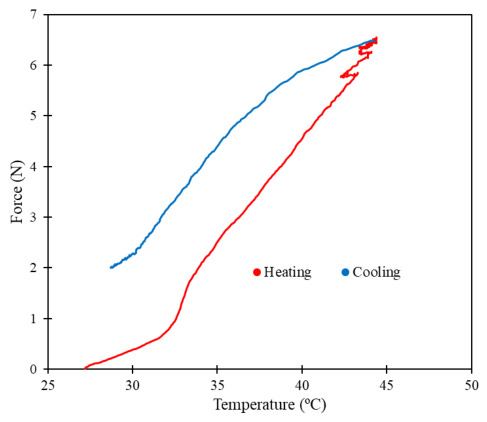
Force exerted by the commercial nitinol as a function of its temperature.

Figure 6 presents the measured force magnitudes of the commercial nitinol as a function of its temperature. The measured force increased with temperature, reflecting the thermally induced martensite-to-austenite phase transformation characteristic of this shape memory alloy. A force magnitude stabilization is expected at a temperature close to 45 °C, the transformation temperature of the commercial nitinol. The oscillation in the measured forces, approximately between 40 °C and 45 °C, probably indicates the beginning of the low-temperature to high-temperature phase transition. The measured force magnitudes are in the same range as those from traction sutures for esophageal elongation in an animal model reported by K. Toczewski et al. [36].

### 3.4. Chemical Composition

**Figure 7:**
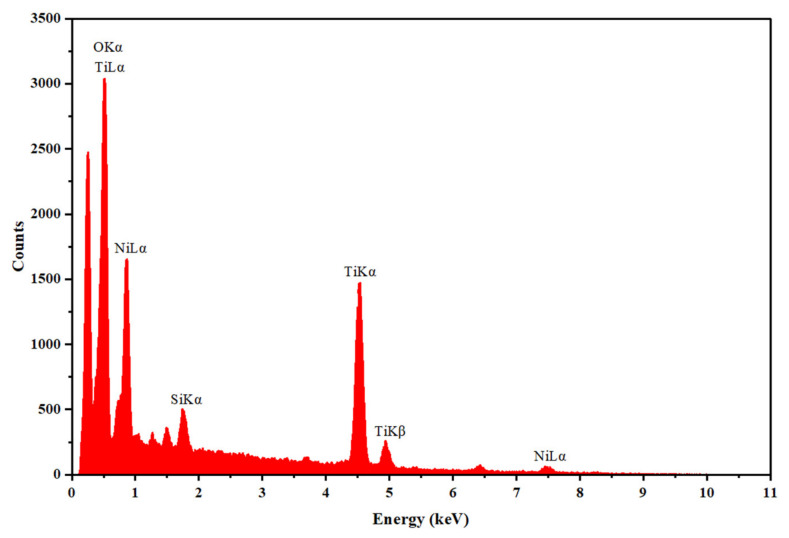
EDS spectrum of the commercial nitinol.

The surface chemical composition of the commercial nitinol was assessed by EDS, with an accelerating voltage of 10 kV. Figure 7 shows the result, confirming Ni (13.15 at.%), Ti (26.73 at.%), and oxygen (56.44 at.%) as the main elemental constituents of the nitinol surface. Minor amounts of other elements were also detected (<2 at.%), attributed to surface contamination or processing residues. Thus, despite the presence of Ni, the surface composition is dominated by Ti and oxygen, which is consistent with the formation of a passive titanium dioxide (TiO_2_) layer commonly reported for NiTi alloys and associated with improved corrosion resistance and biocompatibility [13,37].

### 3.5. FEM Simulation Results

**Figure 8:**
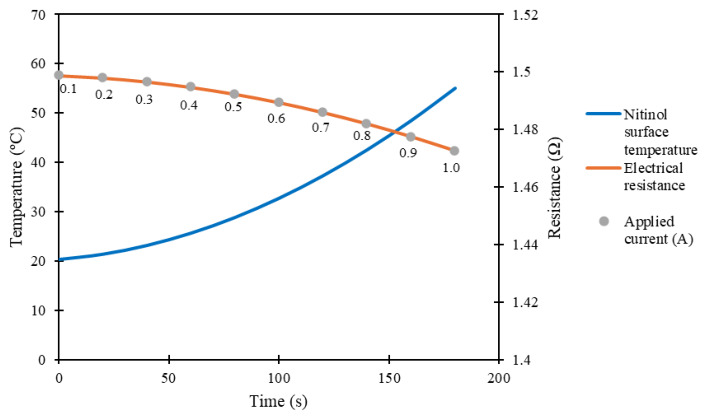
Simulation of Joule heating of the commercial nitinol.

The electrical resistance obtained in the first step of simulations, concerning the commercial nitinol when fully contracted, was 1.409 Ω (16 mm of length), and when fully extended was 1.373 Ω (50 mm of length). Figure 8 shows the obtained temperature and electrical resistance in the second step of the simulations. The simulation results are very close to the ones obtained experimentally, with the overall reached temperatures in the range of ±20–60 °C, and the electrical resistances in the range of ±0.8–1.5 Ω. Slight differences can be explained by the contraction of the commercial nitinol during its heating, which was not considered in the simulations.

### 3.6. Ex Vivo Tests with Thermal Monitoring

**Figure 9:**
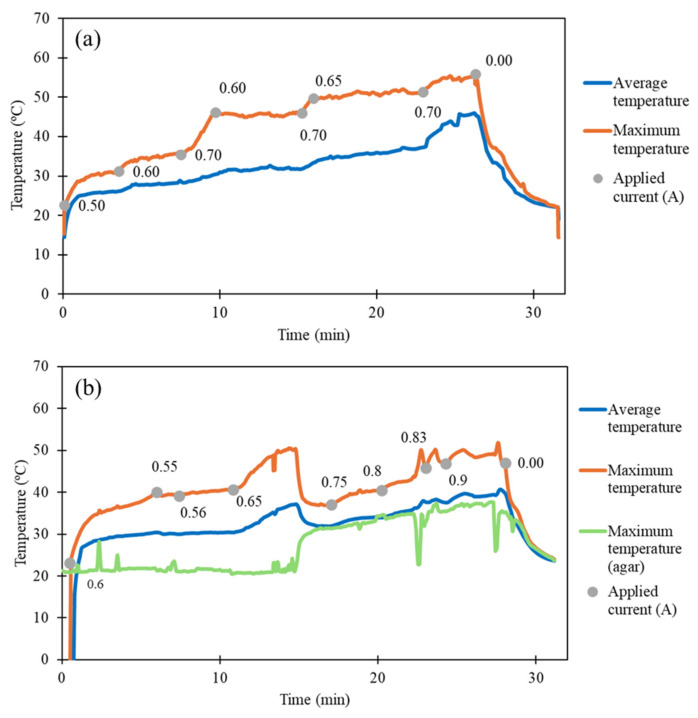
Ex vivo tests with thermal monitoring without agar phantom (**a**) and with an agar phantom (**b**), indicating the temperatures of the commercial nitinol (average and maximum temperature), the temperature of the agar phantom (maximum temperature (agar)), and the applied electrical currents (applied current (A)).

The electrical approach was explored in the ex vivo tests. The electrical current led to the contraction of the commercial nitinol, gradually approximating the esophageal segments. Table 5 and Figure 9 show the results obtained from the ex vivo tests with thermal monitoring.

In the first test (Figure 9a), the average temperature of the nitinol did not exceed 45 °C, but its maximum temperature reached 55 °C. The nitinol’s minimum length was 23 mm, and its final length was 28 mm. In the second test (Figure 9b), the average temperature of the nitinol did not exceed 40 °C, and its maximum temperature reached 50 °C. The minimum length of the nitinol was 25 mm, and the final length was 29 mm. Thus, it can be noted that 5 min after stopping the application of electrical current, the length of the nitinol coil increases by an average of 19%. Thus, nitinol did not return to its initial length, even under the tension of the tissues. The average nitinol final length was lower than 30 mm, the threshold usually considered to define long-gap esophageal atresia. This is a positive result concerning the medical application.

Additionally, during the second test (Figure 9b), an agar phantom (2%, to mimic soft tissue [38]) was used to simulate the heat transfer between the nitinol and the surrounding tissues. Even when the coil reached a maximum temperature of 50 °C, the agar phantom temperature did not exceed 40 °C, which suggests that the risk of thermal damage is minimal for the employed temperatures, which is also a positive result for application in real scenario. Finally, it is also important to note that for a real scenario application, esophagus elongation must be performed gradually (some millimeters per day [39]) to minimize tissue rupture, and thus, the application of electrical current will not need to be continuous.

### 3.7. NiTi Sputtered Thin-Films

NiTi thin-films were deposited by sputtering. NiTi film sputtering deposition is widely recognized as a preferred method due to the precise control of the deposition process and the consistency of the quality of the thin-films [40,41]. The NiTi target (48 at.% Ni and 52 at.% Ti) aims to compensate for the titanium losses commonly observed during sputtering deposition, due to oxide formation and angular flux distributions [42].

**Figure 10:**
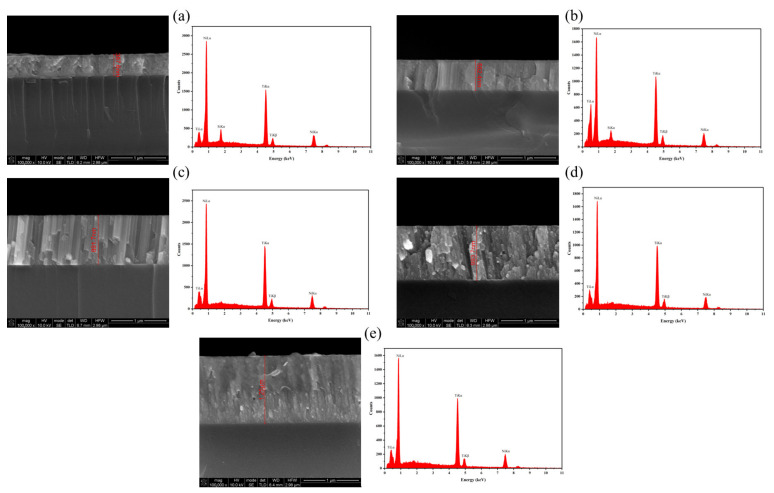
SEM image (cross-section view) and EDS spectrum of the thin-films: 1 (**a**), 2 (**b**), 3 (**c**), 4 (**d**), and 5 (**e**).

The deposited thin-films were analyzed by SEM to assess each film’s thickness. The chemical composition of each thin-film was also assessed by EDS, with an accelerating voltage of 12 kV. Figure 10 and Table 6 show the obtained results, considering the thickness and the main elemental constituents of each thin-film. Some relevant conclusions can be taken from these results: (1) the EDS result from thinner films is affected by the contribution of Si from the substrate; (2) the decrease in the substrate–target distance optimizes the Ti deposition; and (3) for a real scenario application, where it is intended to obtain a NiTi film with a transformation temperature of approximately 40 °C, the process parameters for obtaining thin-film 4 are the most suitable, considering that the Ni content must be slightly higher than 50 at.% [21].

**Figure 11:**
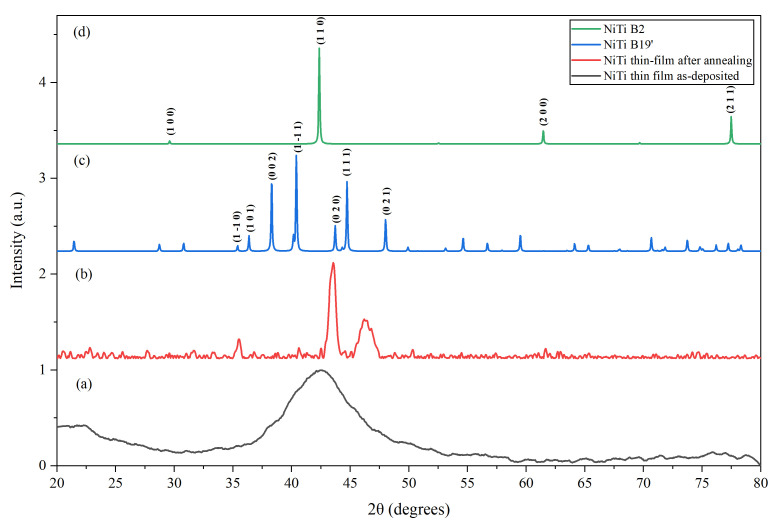
XRD patterns of thin-film 1 (**a**), thin-film 2 (**b**), and simulated diffraction patterns of B19’ (**c**) and B2 (**d**).

The thin-films 1 and 2 were also analyzed by XRD. Figure 11a shows the XRD pattern for thin-film 1. The diffraction pattern exhibits a broad peak centered at approximately 42°, indicating an amorphous structure. This behavior is consistent with a previous report where NiTi thin-film, as deposited, is amorphous [43]. Figure 11b shows the XRD pattern for thin-film 2, which was subjected to an annealing process. The result reveals the appearance of well-defined diffraction peaks characteristic of the monoclinic B19’ martensitic phase at room temperature, confirming crystallization of the NiTi thin-film. This result agrees with the literature, where thermal annealing promotes phase formation and structural ordering in sputtered NiTi thin-films [37,43,44,45]. To support phase identification, the experimental pattern (Figure 11b) was compared with simulated diffraction patterns for the B19’ (martensite) and B2 (austenite) phases (Figure 11c,d), obtained from crystallographic information files available in the Crystallography Open Database [46]. The experimental peak positions show good overall agreement with the B19’ phase. However, slight deviations are observed, which may be attributed to residual stresses, preferred orientation, and compositional variations relative to the NiTi structure used in the simulated patterns.

The preliminary research work presented in this section describes the developments towards the final goal of microfabricating a nitinol spring with dimensions (nitinol spring with a wire thickness of a few micrometers) and transformation temperature (±40 °C) more suitable for solving long-gap EA in newborns. Procedures based on a variety of microfabrication technologies (sputtering deposition, lithography, etching, and annealing processes) are expected to be fundamental for the future development of a solution for this medical condition. The application of those microfabrication technologies for the fabrication of nitinol-based medical devices is also reported in the literature [47].

## 4. Conclusions

This research proposes a new and innovative approach for repairing long-gap EA by using nitinol to achieve controlled esophageal elongation. Here, a first proof-of-concept with a commercial solution is presented. Several experimental tests and simulations were performed to characterize commercial nitinol, including the application of an electrical current to promote its heating and contraction, tensile tests, and chemical composition analysis. The experimental electrical tests were in accordance with the simulation results, considering the nitinol reached temperatures, electrical resistances, and the measured Seebeck coefficient. The obtained contraction forces and chemical composition are also in accordance with the literature. The ex vivo tests using porcine esophagus tissues and an agar phantom further demonstrated that the commercial nitinol did not return to its initial length, even when tensioned by the esophageal segments. Moreover, no thermal damage was observed during the ex vivo tests. All the results are key aspects for the suitability of this approach and give promising signs for the future research of this solution in a clinical environment. Finally, preliminary work was also presented concerning the future intention of microfabricating a nitinol spring for EA repair in newborns, with more suitable dimensions and transformation temperature. The preliminary results of the SEM, EDS, and XRD analyses of sputtering-deposited NiTi thin-films are also encouraging for future directions of this nitinol-based solution for solving a medical condition.

## Figures and Tables

**Table 1:** Geometry parameters for the commercial nitinol used in the simulations.

Spring	Form	Number of Turns	Major Radius (mm)	Minor Radius (mm)	Axial Pitch Fully Extended (mm)	Axial Pitch Fully Contracted (mm)
Spring 0.75 mm	Helical	21	3.25	0.375	2.5	0.75

**Table 2:** Physical parameters of the nitinol, considering the martensite phase.

Parameter	Value (Martensite Phase)
Electrical conductivity	1,041,341 S/m
Relative permittivity	1
Density	6500 Kg/m^3^
Thermal conductivity	14 W/(mK)
Heat capacity at constant pressure	693 J/(KgK)
Relative permeability	~1
Young’s modulus	30 GPa
Poisson’s ratio	0.33

**Table 3:** Physical parameters of the nitinol, considering the austenite phase.

Parameter	Value (Austenite Phase)
Initial electrical conductivity	1 × 10^6^ S/m
Temperature coefficient of resistivity	−5 × 10^−4^ K^−1^
Relative permittivity	1
Density	6500 Kg/m^3^
Thermal conductivity	28 W/(mK)
Heat capacity at constant pressure	911 J/(KgK)
Relative permeability	~1
Young’s modulus	70 GPa
Poisson’s ratio	0.33

**Table 4:** Deposition parameters of the NiTi thin-films.

Thin-Film	Vacuum Pressure (mbar)	Argon Gas Flow (sccm)	Working Pressure (mbar)	Power (W)	Substrate–Target Distance (cm)
1	7.5 × 10^−6^	10	4.8 × 10^−3^	150	11.3
2	7.5 × 10^−6^	15	8.1 × 10^−3^	120	11.3
3	7.6 × 10^−6^	10	7.1 × 10^−3^	120	11.3
4	8.4 × 10^−6^	10	8.1 × 10^−3^	120	7.6
5	9.2 × 10^−6^	15	7.8 × 10^−3^	120	7.6

**Table 5:** Obtained results for the ex vivo tests with the commercial nitinol and the esophageal tissues.

Test Number	Time for Maximum Contraction (min:s)	Minimum Length of the Nitinol (mm)	Final Length of the Nitinol (mm)
1	26:19	23	28
2	28:03	25	29

**Table 6:** SEM and EDS results for the different deposited NiTi thin-films.

Thin-Film	Thickness (nm)	Deposition Rate (nm/s)	Ni (at.%)	Ti (at.%)	Si (at.%)
1	388	0.35	50.04	44.72	5.23
2	562	0.33	48.34	47.92	3.74
3	892	0.34	51.38	48.62	-
4	994	0.41	50.16	49.84	-
5	1250	0.52	49.73	50.27	-

## Data Availability

The original contributions presented in this study are included in the article. Further inquiries can be directed to the corresponding author.

## Abbreviations

The following abbreviations are used in this manuscript:

EA	Esophageal atresia
TF	Tracheoesophageal fistula
EDS	Energy dispersive X-ray spectroscopy
SEM	Scanning electron microscopy
XRD	X-ray diffraction
